# Query-Driven Retinal Layer Segmentation in OCT Using Cross-Attentive Feature Learning

**DOI:** 10.3390/diagnostics16111697

**Published:** 2026-05-31

**Authors:** Nebras Sobahi, Salih Taha Alperen Özçelik, Orhan Atila, Abdulkadir Sengur, Muhammed Halil Akpınar

**Affiliations:** 1 Department of Electrical and Electronics Engineering, Faculty of Engineering, King Abdulaziz University, Jeddah 21589, Saudi Arabia; nsobahi@kau.edu.sa; 2 Department of Electrical-Electronics Engineering, Faculty of Engineering, Bingol University, 12000 Bingol, Türkiye; 3 Department of Electrical-Electronics Engineering, Faculty of Technology, Firat University, 23100 Elazig, Türkiye; oatila@firat.edu.tr (O.A.); ksengur@firat.edu.tr (A.S.); 4 Department of Electronics and Automation, Vocational School of Technical Sciences, Istanbul University-Cerrahpasa, 34098 Istanbul, Türkiye; mhakpinar@iuc.edu.tr

**Keywords:** OCT, retinal layer segmentation, transformer, query-based learning, cross-attention, medical image segmentation

## Abstract

**Background/Objectives**: Retinal layer segmentation in optical coherence tomography (OCT) is essential for the diagnosis and monitoring of retinal diseases such as age-related macular degeneration (AMD) and diabetic macular edema (DME). Although deep learning methods have achieved strong performance, most rely on dense pixel-wise predictions and often struggle to preserve anatomical consistency, particularly in regions with low contrast or structural deformation. This study aims to address these limitations by introducing a query-based segmentation framework that explicitly models retinal layer structure. **Methods**: In this paper, we propose the RetiQueryNet architecture that employs encoding of retinal layers in the form of query embeddings with the use of cross attention to interact with pixel level features encoded by a transformer based encoder. The architecture integrates multi-scale features through a compact query-driven decoder with modest additional computational overhead. Normalization and resizing of OCT images preceded their usage as inputs, while the layer labels were converted to multi-class segmentation maps. In the training process, we used loss function with combination of cross entropy loss and Dice loss. Our model performance was compared with multiple state-of-the-art models such as U-Net, DeepLabV3, FPN, MANet and SegFormer, while performance metrics were Dice, IoU and mean surface distance (MSD). **Results**: RetiQueryNet was able to attain a mean Dice score of 0.934 ± 0.0046 and outperformed all baseline models on the main performance measures. Improvements were particularly evident in challenging retinal layers such as IBRPE and OBRPE, where boundary ambiguity is high. It should be noted that RetiQueryNet had a relatively lower MSD value, meaning that the predicted boundaries were more accurate. Furthermore, visual observations suggest that the approach generated smooth and coherent segmentations. **Conclusions**: The findings demonstrate that query-based modeling offers a viable approach to pixel-wise segmentation. In particular, by making use of structural priors in the form of learnable queries, RetiQueryNet improves not only segmentation accuracy but also anatomical consistency. Query-based modeling appears to be an exciting area for retinal image segmentation that could potentially be applied to other applications in medical image segmentation.

## 1. Introduction

Over the past two decades, optical coherence tomography (OCT) has become an indispensable tool in ophthalmology, allowing one to image retinal layers with micrometer-level resolution. Within daily clinical practice, the thickness, continuity, and integrity of retinal layer boundaries are critical for the management of diseases like AMD and DME. Thus, accurate segmentation of retinal layers is necessary not only as a technical step but rather for the quantitative analysis of retinas and proper patient management [1].

Although the technique has high medical significance, segmenting the retinal layers using OCT scans is still considered difficult. The layers in an OCT scan are often thin, display low contrast and ambiguity at local scales. In addition, speckle noise and shadowing are common in OCT images, along with disease-specific changes to the structure. Thus, traditional image analysis pipelines are increasingly being replaced by deep learning methods, which first used encoder–decoder CNNs and later Transformer networks. According to recent reviews, such models perform much better, but results strongly depend on data and assessment criteria [1]. This development in retinal image analysis is part of a broader expansion of artificial intelligence across healthcare. Recent studies have demonstrated the value of machine learning in diagnostic support, risk prediction, and medical image interpretation in several clinical domains. Examples include AI-based diagnostic and predictive modeling for polycystic ovary syndrome, neural-network-based analysis of noisy MRI images for cancer-related diagnosis, and explainable machine learning approaches for the early detection of type 2 diabetes [2,3,4]. These studies illustrate the growing role of data-driven methods in improving diagnostic consistency and supporting complex clinical decision-making.

An additional problem associated with this issue is that research in the field has developed faster than the benchmarks used to evaluate it. Many works demonstrate highly accurate overlap-based measures; however, the research was conducted with different datasets characterized by dissimilarities in disease prevalence, labeling rules, scanner, and metric choice. The domain shift problem also remains relevant. One of the latest studies exploring the application of foundation models for retinal layer segmentation shows decreased performance due to changes in the scanner and its parameters despite identical tasks [5].

The existence of OCT5k plays an important role in solving this problem to some extent. This dataset provides 5016 pixel-wise manual annotations for 1672 OCT B-scans, along with multiple-grader annotations, boundary annotations, and examples of patients with AMD, DME, and normal cases [6]. Such additional layers of complexity increase the realness of the benchmark, but they also increase the difficulty of the problem. Now, models can no longer deal with just one homogeneous group of samples, as they have to account for the structural differences between various diseases, while still keeping their layer boundaries intact. In this context, simple pixel-level segmentation may not be enough, since what really matters here is to distinguish separate structures of retina layers.

Transformers benefit in this context due to their improved ability to model long-range dependencies and multi-scale contextual relationships, outperforming several previous CNN models. SegFormer serves as a good example for such an architecture that shows impressive results. By leveraging the hierarchical Transformer architecture and combining it with an efficient decoder, SegFormer has proven itself as an effective segmentation backbone [7]. However, traditional segmentation decoders still view output generation as a dense pixel labeling task. For retinal OCT, this view can be limiting. The target structures are not arbitrary semantic regions. They are ordered, thin, and anatomically coupled layers. A decoder that models each layer as an explicit target may therefore be better suited to the problem than a generic dense prediction head.

Motivated by this gap, we propose RetiQueryNet, a query-based model for retinal layer segmentation in OCT. Instead of relying only on a conventional dense decoder, RetiQueryNet represents retinal layers through layer-aware queries and learns to recover their masks in a more structured way. The goal is simple: keep the strong representation power of a modern Transformer backbone while improving layer discrimination and boundary consistency in challenging regions. This design is particularly relevant on OCT5k, where multi-disease variability and fine anatomical detail make the task more demanding [6].

This work makes three main contributions. First, we introduce RetiQueryNet, a query-based architecture tailored to retinal layer segmentation in OCT. Second, we evaluate the proposed model against a set of strong baseline networks under a consistent experimental protocol. Third, we report both overlap-based and boundary-sensitive metrics, allowing us to assess not only region agreement but also boundary quality, which is critical for retinal layer analysis [1,6]. The remainder of this paper is organized as follows. Section 2 describes the dataset, preprocessing steps, and the proposed method. Section 3 presents the quantitative and qualitative results, including ablation, efficiency, sensitivity, and failure-case analyses. Section 4 discusses the findings and limitations, and Section 5 concludes the paper.

Studies on retinal layer segmentation have focused on improving segmentation performance and classification reliability by applying various methods. The methods used include 3D volumetric approaches involving nnU-Net models, adapted versions of the U-Net++ model that involve multiclass segmentation of retinal layers and fluids, hybrid approaches combining both InceptionNet and U-Net for specific clinical purposes like macular hole segmentation, and the use of foundation models such as SAMs (Segment Anything Models).

Deep learning is the dominant method of OCT segmentation in retinal images. The early developments have mainly relied on convolutional encoder–decoder models, where U-Net and variations of this model have played an important role. These models remain effective baselines because of the ability of skip connections to capture local information alongside contextual information at multiple scales. With time, the family of models has evolved as a result of incorporating residual blocks, attention, and efficient designs that facilitate the task of thin retinal layers and OCTs. LightReSeg is an example of such a recent development. It combines a lightweight encoder–decoder design with global reasoning and reports strong performance on retinal layer segmentation while keeping the model efficient [8]. More broadly, recent reviews agree that CNN-based methods still offer a reliable foundation, particularly when the dataset is limited or when stable training is a priority [1,9].

Transformer-based methods were introduced to address some of the known limits of purely convolutional models. In OCT images, long-range context matters because retinal layers extend horizontally, and their local appearance may change under pathology. Hierarchical vision transformers and hybrid CNN-Transformer networks have therefore become increasingly popular. SegFormer is one of the most influential examples because it provides strong multi-scale representations with a relatively simple decoder [7]. More recent OCT studies have followed this direction and reported competitive or improved performance with transformer-enhanced designs, including self-attention CNN hybrids, SegFormer-based pipelines, and Swin-style architectures [10,11,12].These models often improve contextual modeling, but most of them still rely on dense prediction heads that treat the segmentation task mainly as pixel-wise classification. That choice is effective, yet it may underuse the structured nature of retinal layer anatomy. Query-based visual reasoning has also influenced modern segmentation architectures. DETR introduced learnable queries for set-based prediction, while MaskFormer reformulated segmentation as a mask-classification problem in which a fixed set of queries predicts masks and their semantic labels [13,14]. RetiQueryNet follows the broader idea of query-driven prediction, but it differs from MaskFormer in both design and purpose. MaskFormer was developed as a general-purpose framework for semantic and panoptic segmentation, whereas RetiQueryNet is tailored to retinal layer segmentation in OCT. Instead of predicting generic mask proposals, RetiQueryNet uses anatomically indexed layer queries, where each query is directly associated with one retinal target class. These queries interact with fused OCT features through a compact cross-attention module and are then converted into class-specific retinal masks through structured decoding. This formulation is designed to reflect the ordered and layer-specific nature of retinal anatomy rather than to solve a generic scene segmentation problem.

Another line of work has focused on improving generalization, efficiency, or structural consistency rather than only increasing overlap scores. Foundation-model-based studies have shown that strong pretrained representations can help retinal layer segmentation, especially when the goal is to transfer across scanners or imaging settings [5]. Other recent work has explored universal segmentation settings, where different datasets with different layer granularities are handled within a shared framework [15]. At the same time, survey papers continue to point out that retinal OCT segmentation is still affected by dataset fragmentation, inconsistent annotation protocols, and the limited use of boundary-sensitive evaluation [1,9]. The importance of this problem is evident due to the fact that higher Dice values do not always indicate proper separation of layers, especially those that lie deep inside the retina where small misalignments can cause considerable problems.

Based on these findings, the mentioned studies suggest two clear trends. First, strong backbones are now available and can produce high-quality retinal OCT segmentation. Second, the decoder side remains a meaningful place for improvement, especially when the goal is to better reflect anatomical structure rather than simply assign labels densely. Our work follows this second direction. Instead of introducing a heavier backbone, we focus on a query-based decoding strategy that is better aligned with the ordered and layer-specific nature of retinal OCT segmentation. Recent studies have also explored OCT segmentation under different clinical targets and dataset configurations. For example, Heine et al. [16] proposed a 3D nnU-Net framework for volumetric retinal analysis and reported a mean Dice score of 0.907 on a large institutional dataset with multiple anatomical and pathological structures. Ndipenoch et al. [17] evaluated fluid segmentation performance across different OCT sources using a modified nnU-Net architecture and achieved a mean Dice of 0.823 on the RETOUCH dataset.

More recent datasets such as RVO-ME [18] have introduced dual-task settings for lesion segmentation and detection, where U-Net++ based approaches reported lower Dice scores (around 0.685), highlighting the increased difficulty of pathology-driven segmentation tasks. Similarly, Daanouni et al. [19] proposed a modified U-Net++ architecture for multi-class segmentation of retinal layers and fluids, achieving Dice scores around 0.91 on the AROI dataset.

Other studies have focused on specific clinical targets such as macular hole segmentation. Herath et al. [20] reported high Dice scores (0.9672) using hybrid architectures combining InceptionNetV4 and U-Net, while recent work based on Segment Anything models (SAM2 and MedSAM2) demonstrated strong generalization across OCT datasets, with Dice scores ranging between 0.88 and 0.91 depending on the dataset [21].

While these models show impressive performances, they often aim to segment diverse targets, including fluid-filled spaces, abnormalities, or even gross anatomical structures. On the other hand, retinal layer segmentation demands accurate modeling of well-defined, organized anatomical layers, and is hence a significantly different task from these problems.

## 2. Materials and Methods

### 2.1. Dataset

**Figure 1:**
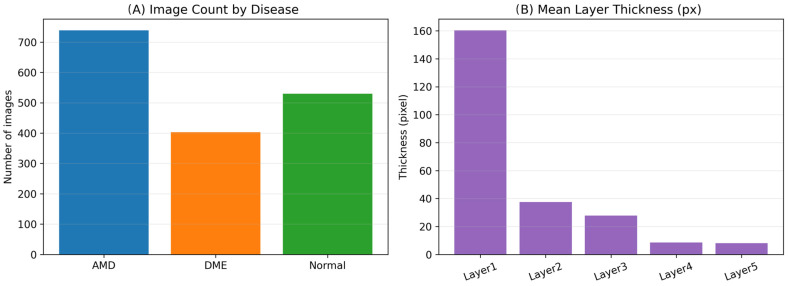
Overview of the OCT5k subset used in this study. (**A**) Number of OCT B-scans in each disease group. (**B**) Mean thickness of the five annotated retinal layers in pixels. The figure highlights both disease imbalance and substantial variation in layer thickness, which together increase the difficulty of retinal layer segmentation.

The proposed RetiQueryNet model was evaluated based on OCT5k dataset, which is a publicly available benchmark dataset for retinal OCT image processing. In this work, we used a manually labeled portion of the dataset aimed at segmenting retinal layers. The portion consists of 1672 OCT B-scan images from 60 patients divided into three groups: AMD, DME, and Normal. It should be mentioned that all images have a unified resolution of 512 × 512 pixels. It is known that there are five retinal layers segmented by this dataset. The size of the layers varies significantly, so one layer is much larger than others. Thus, as it can be seen in Figure 1, the size of the first layer exceeds those of other layers considerably, whereas the last layers are relatively narrow. This makes segmentation rather complicated because small displacements in narrow layers become noticeable. Furthermore, the structural variation between different diseases becomes noticeable as well. It can be noted that in DME group of images, the average thickness of middle layers is larger compared to AMD and Normal groups.

These properties make OCT5k a suitable and realistic benchmark for retinal layer segmentation. The model must not only segment multiple anatomically ordered layers, but also remain robust to class imbalance and morphology changes across disease groups. Statistical information regarding class distributions and layer thicknesses for the OCT5k dataset is given in Table 1.

### 2.2. Preprocessing and Data Split

All OCT B-scans were processed using a unified input pipeline. Since every image in the manually annotated subset has a fixed spatial size of 512 × 512 pixels, no aspect-ratio correction was required. The training and evaluation scripts were configured to use this native resolution throughout the experiments.

**Figure 2:**
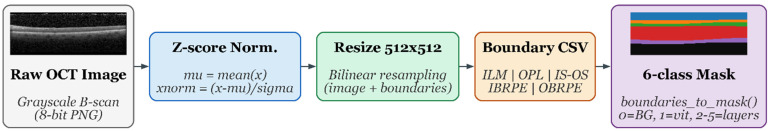
Preprocessing pipeline used in this study.

As illustrated in Figure 2, each OCT image first undergoes z-score normalization, where the intensity values are standardized using the mean and standard deviation of the image. This step reduces intensity variations across scans and improves training stability. The normalized images are then resized to a fixed resolution of 512 × 512 using bilinear interpolation. Importantly, the same resizing operation is applied consistently to both the image and its corresponding structural annotations.

Each OCT B-scan is first normalized using z-score normalization, followed by resizing to a fixed resolution of 512 × 512 pixels. Retinal layer boundaries provided as CSV annotations are then converted into a dense multi-class segmentation mask, where each class corresponds to a specific retinal layer. The pipeline ensures a constant input representation and enables supervised learning for the segmentation model. The database provides retinal layers as boundaries represented using CSV files. These boundaries represent important anatomical boundaries, namely the Inner Limiting Membrane (ILM), Outer Plexiform Layer (OPL), Inner Segment–Outer Segment junction (IS-O), Inner Boundary of the Retinal Pigment Epithelium (IBRPE), and Outer Boundary of the Retinal Pigment Epithelium (OBRPE). In the final preprocessing step, these boundary annotations are converted into dense segmentation masks using a deterministic mapping function. The resulting masks contain six classes, including background and five retinal layers, which are used as targets during training.

To avoid data leakage, we adopted a case-based split rather than an image-level random split. Separate dataset objects were used for training and evaluation. The training subset included data augmentation, while the validation and test subsets used only deterministic preprocessing. After splitting, validation and test sets were reconstructed from the evaluation dataset to ensure that no augmented samples appeared during model selection or final testing. This guarantees that images from the same case are not distributed across different splits. During training, data augmentation was applied only to the training subset in order to improve robustness to moderate appearance and geometric variations. The augmentation pipeline included random horizontal flipping with a probability of 0.5, affine transformations with random scaling in the range of 0.9–1.1, translation up to 5% of the image size, and in-plane rotation up to ±10° with a probability of 0.4. In addition, random brightness and contrast perturbations were applied with a probability of 0.3. All transformed images and their corresponding segmentation masks were spatially aligned throughout augmentation. No augmentation was applied to the validation or test subsets.

All experiments were conducted using three random seeds (42, 123, and 456) to reduce variance due to data partitioning. For each seed, the same protocol was applied to all models. The dataset was divided into 70% training, 15% validation, and 15% testing at the case level. Models were trained for 50 epochs using the AdamW optimizer with an initial learning rate of 1 × 10^−3^ and a cosine annealing schedule. The best model was selected based on the validation Dice score and evaluated on the held-out test set.

### 2.3. Proposed Method: RetiQueryNet

**Figure 3:**
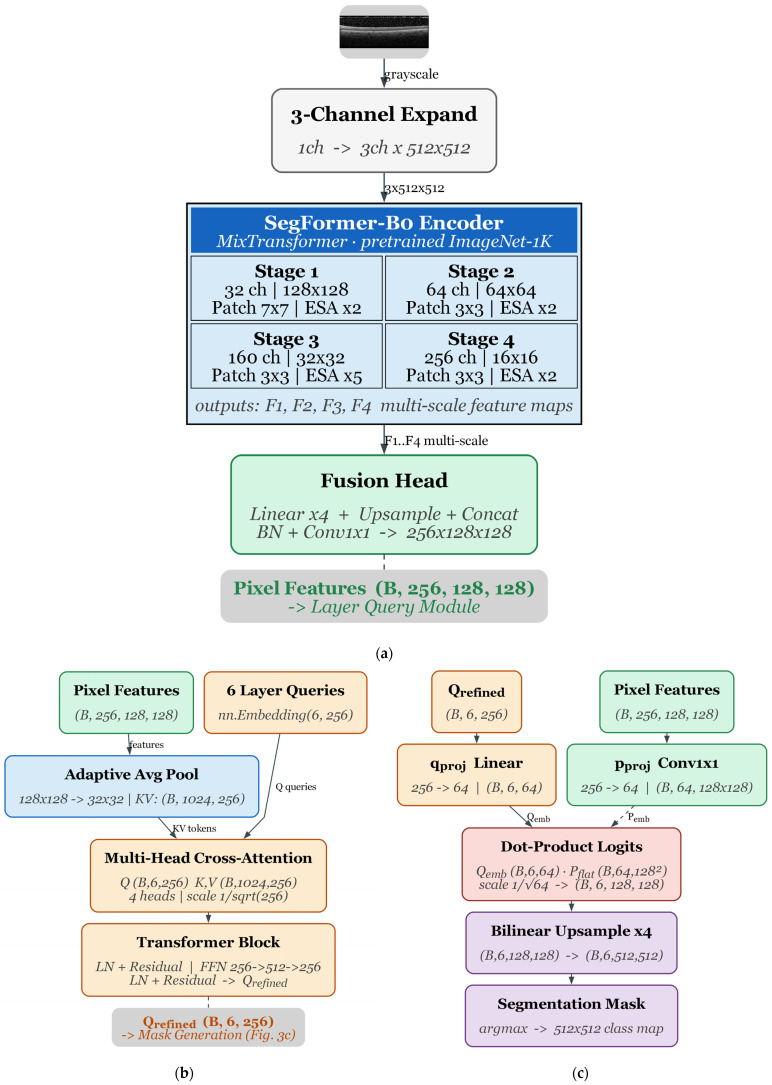
Overall architecture of RetiQueryNet. (**a**) Multi-scale feature extraction using the SegFormer-B0 backbone. (**b**) Query-based interaction module, where learnable layer queries attend to pixel-level features through cross-attention. (**c**) Mask generation stage, where query embeddings are projected into segmentation masks via a shared embedding space.

We propose RetiQueryNet, a query-based retinal layer segmentation model designed to better capture the structured and ordered nature of OCT retinal anatomy. Instead of treating segmentation as a purely dense pixel classification problem, the model represents each retinal layer as an explicit target through a set of learnable queries [14].An overview of the full architecture is shown in Figure 3, where the pipeline is divided into three main stages: feature extraction (Figure 3a), query-based interaction (Figure 3b), and mask generation (Figure 3c). Although the proposed model belongs to the broader family of query-based segmentation methods, it is not a direct adaptation of MaskFormer. RetiQueryNet does not use a generic mask-classification pipeline with a set of free-form mask proposals. Instead, it defines a small number of layer-specific queries that are anatomically tied to the retinal classes of interest. The decoder therefore serves as a task-specific structural reasoning module, enabling direct interaction between retinal layer queries and OCT feature maps with limited computational overhead.

The model takes a single-channel OCT B-scan as input. Since the backbone is initialized from ImageNet pretraining, the grayscale image is first expanded to three channels. Let the input image be denoted as $x\in R^{1\times H\times W}$. After channel expansion, it becomes $x^{\prime }\in R^{3\times H\times W}$, which allows compatibility with standard pretrained Transformer encoders.

#### 2.3.1. Multi-Scale Feature Extraction

As illustrated in Figure 3a, we adopt a SegFormer-B0 encoder as the backbone. The encoder produces hierarchical feature maps at multiple resolutions:

$$\left\{F_{1},F_{2},F_{3},F_{4}\right\}$$

where each $F_{i}$ captures information at a different spatial scale. These features are then fused using a lightweight decoder that aligns them to a common resolution and aggregates them into a unified representation:

$$F_{\mathrm{fused}}=\mathrm{Fuse}(F_{1},F_{2},F_{3},F_{4})$$

The resulting feature map has a fixed dimensionality and spatial size, forming dense pixel-level features:

$$F\in R^{B\times C\times H^{\prime }\times W^{\prime }}$$

In our implementation, this corresponds to C=256 and $H^{\prime }=W^{\prime }=128$. These features serve as the shared representation for all subsequent layer predictions.

#### 2.3.2. Query-Based Layer Representation

Instead of directly predicting class labels per pixel, we introduce a set of layer queries that explicitly represent each retinal layer. As shown in Figure 3b, we define a learnable embedding matrix:

$$Q\in R^{N\times C}$$

where N=6 corresponds to the number of output classes (background + five retinal layers). In the present study, the number of queries is set equal to the number of segmentation classes, namely one background class and five retinal layer classes. However, this formulation is not restricted to a fixed anatomical granularity. For a dataset containing a different number of retinal layers or a revised annotation scheme, the query set can be resized accordingly by changing the number of learnable embeddings and the corresponding output channels. The remaining architecture, including the cross-attention mechanism and mask generation strategy, remains unchanged. Each query is intended to capture the global representation of a specific layer. The layer queries are implemented as trainable embedding vectors. At initialization, each query embedding is randomly sampled from a zero-mean normal distribution with a standard deviation of 0.02. These embeddings are not derived from external pretraining; instead, they are optimized jointly with the backbone and decoder during end-to-end training. This design allows the model to learn layer-specific structural representations directly from the OCT segmentation task.

The interaction between pixel features and layer queries is modeled through a cross-attention mechanism, which allows each query to selectively gather relevant information from the feature map. This produces refined query representations:

$$Q_{\mathrm{refined}}=\mathrm{Transformer}(Q,F)$$

These refined queries encode both global context and layer-specific information, enabling the model to distinguish between anatomically adjacent structures more effectively.

#### 2.3.3. Mask Generation

The final segmentation masks are generated by projecting both the refined queries and pixel features into a shared embedding space, as illustrated in Figure 3c. Specifically, we apply linear projections:

$$Q^{\prime }=W_{q}Q_{\mathrm{refined}},F^{\prime }=W_{p}F$$

where $W_{q}$ and $W_{p}$ are learnable projection matrices. The segmentation logits are then computed using a scaled dot-product operation:

$$M=Q^{\prime }\cdot F^{\prime }$$

This produces a set of class-wise activation maps, which are upsampled to the original image resolution:

$$\hat{Y}=\mathrm{Upsample}(M)$$

Finally, the predicted segmentation is obtained via an argmax operation over classes:

$$Y=arg\;\underset{c}{max}{\hat{Y}}_{c}$$

This formulation allows each query to generate its own segmentation mask, while the shared feature space ensures consistency across layers. RetiQueryNet combines the strong representation power of a Transformer model as the backbone with a clear decoder mechanism. With the modeling of each retinal layer using queries, a more systematic approach can be used as compared to the dense decoder heads of traditional architectures.

### 2.4. Layer Query Cross-Attention Module

**Figure 4:**
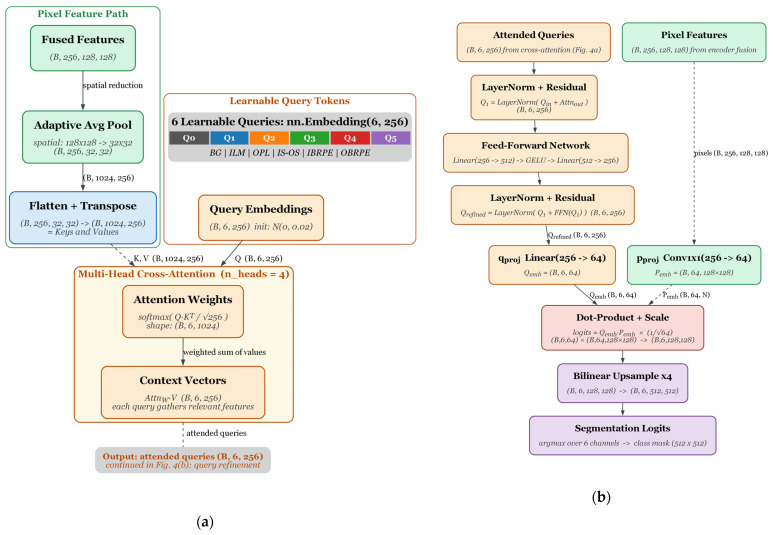
Layer Query Cross-Attention module. (**a**) Cross-attention interaction between pixel features and layer queries, where queries selectively attend to relevant spatial regions. (**b**) Transformer refinement block with residual connections and feed-forward layers, producing refined query embeddings for mask prediction.

In order to capture the relationship between the retinal layers and pixel-wise features more directly, we propose the Layer Query Cross Attention, illustrated in Figure 4. This layer plays a crucial role in RetiQueryNet by converting general pixel-wise features into layer-aware features [13].

As shown in Figure 4a, the module operates on two inputs: the fused pixel features and a set of learnable layer queries. The fused feature map

$$F\in R^{B\times C\times H\times W}$$

is first spatially reduced using adaptive average pooling to obtain a compact representation:

$$F_{\mathrm{pool}}\in R^{B\times C\times H_{p}\times W_{p}}$$

This step reduces computational cost while preserving global context. The pooled feature map is then flattened and transposed to form key and value tensors:

$$K,V\in R^{B\times N\times C}$$

where $N=H_{p}\cdot W_{p}$ denotes the number of spatial tokens.

In parallel, we define a set of learnable query embedding’s:

$$Q\in R^{B\times L\times C}$$

where L=6 corresponds to the number of segmentation classes (background + five retinal layers). Each query is intended to capture the global representation of a specific layer.

#### 2.4.1. Cross-Attention Interaction

The interaction between queries and pixel features is computed using multi-head cross-attention:

$$\mathrm{Attn}(Q,K,V)=\mathrm{softmax}\left(\frac{QK^{T}}{\sqrt{C}}\right)V$$

This operation allows each query to selectively attend to relevant spatial regions. The output consists of attended query representations:

$$Q_{\mathrm{attn}}\in R^{B\times L\times C}$$

Each query aggregates context from the feature map in a layer-specific manner. Unlike dense decoders, this formulation introduces an explicit correspondence between queries and anatomical layers.

#### 2.4.2. Transformer Refinement

As shown in Figure 4b, the attended queries are further refined using a Transformer-style block. This includes a residual connection followed by layer normalization:

$$Q_{1}=\mathrm{LayerNorm}(Q+Q_{\mathrm{attn}})$$

The refined queries are then passed through a feed-forward network:

$$\mathrm{FFN}(Q_{1})=W_{2}\cdot \sigma (W_{1}Q_{1})$$

where σ denotes the GELU activation. A second residual connection produces the final query representation:

$$Q_{\mathrm{refined}}=\mathrm{LayerNorm}(Q_{1}+\mathrm{FFN}(Q_{1}))$$

This refinement step improves the expressive capacity of each query while preserving stability through residual connections.

#### 2.4.3. Projection and Mask Generation

The refined queries and pixel features are projected into a shared embedding space:

$$Q_{\mathrm{emb}}=W_{q}Q_{\mathrm{refined}},F_{\mathrm{emb}}=W_{p}F$$

where $W_{q}$ and $W_{p}$ are learnable projection layers. The segmentation logits are computed using a scaled dot-product:

$$\mathrm{logits}=\frac{Q_{\mathrm{emb}}\cdot F_{\mathrm{emb}}}{\sqrt{d}}$$

This produces class-wise activation maps:

$$\in R^{B\times L\times H\times W}$$

which are then up-sampled to the original resolution and converted into final predictions.

Overall, this module enables layer-specific reasoning by allowing each query to gather and refine information independently. By combining global attention with structured decoding, the model can better preserve thin boundaries and maintain consistency across adjacent retinal layers.

### 2.5. Loss Function and Training Strategy

**Figure 5:**
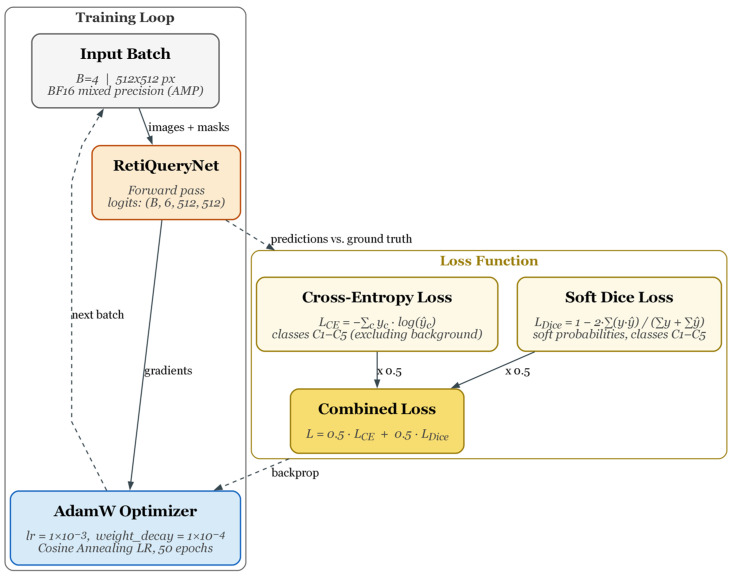
Training pipeline of RetiQueryNet.

To train RetiQueryNet effectively under class imbalance and thin-layer constraints, we adopt a composite loss function together with a stable optimization scheme. The overall training pipeline is illustrated in Figure 5.

Each input batch is used to obtain logits of segmentations through the neural network. Next, the predictions are checked against the masks provided using the combined loss function consisting of cross-entropy and soft Dice functions. The obtained gradients are used to update the weights of the model using AdamW optimizer and cosine learning rate schedule.

#### 2.5.1. Loss Function

The two main issues in retinal layer segmentation are (i) severe class imbalance due to unequal thickness of different layers, and (ii) increased sensitivity to errors in the case of thin layers. In order to solve the above two issues, cross-entropy loss and soft dice loss are combined:

$$L_{CE}=-\sum_{c}y_{c}log({\hat{y}}_{c})$$

where $y_{c}$ and ${\hat{y}}_{c}$ denote the ground truth and predicted probability for class c, respectively.

The Dice loss focuses on region overlap and helps stabilize training for small structures:

$$L_{Dice}=1-\frac{2\sum (y\cdot \hat{y})}{\sum y+\sum \hat{y}}$$

This formulation is particularly useful for retinal layers with limited spatial extent, where cross-entropy alone may be insufficient [22].

The final loss is defined as a weighted combination:

$$L=\lambda L_{CE}+(1-\lambda )L_{Dice}$$

In our experiments, we use λ=0.5, giving equal importance to both terms. This balance allows the model to learn both accurate class assignments and consistent layer regions.

#### 2.5.2. Training Setup

As shown in Figure 5, training is performed in a standard supervised manner. Each batch consists of OCT images and their corresponding segmentation masks. The model produces class-wise logits:

$$\hat{Y}\in R^{B\times 6\times 512\times 512}$$

which are compared against the ground truth masks to compute the loss.

We used the AdamW optimizer with a weight decay of 1 × 10^−4^. The initial learning rate was set to 1 × 10^−3^ and updated using a cosine annealing schedule throughout training. All baseline models and RetiQueryNet were trained for 50 epochs with a batch size of 4 under the same patient-level data split protocol. Mixed precision training was used to reduce memory consumption and accelerate optimization. The best checkpoint for each run was selected according to the validation Dice score and then evaluated on the held-out test set. The detailed training configuration used in all comparative experiments is summarized in Table 2.

### 2.6. Statistical Analysis

To complement the multi-seed performance reporting, an exploratory paired statistical comparison was performed between RetiQueryNet and the strongest baseline model, SegFormer-B0. The analysis was based on the three matched experimental runs obtained using the same predefined seeds (42, 123, and 456), where both models were evaluated under the corresponding identical data split for each seed. Paired *t*-tests were applied to the seed-wise mDice, mIoU, and mean MSD values. In addition, paired effect sizes were calculated using Cohen’s d_z_, together with 95% confidence intervals for the mean paired differences. Because the analysis is based on only three paired runs, the resulting p-values are interpreted as supportive evidence of consistency across repeated experiments rather than as definitive population-level inference.

### 2.7. External Validation Protocol

To further examine the transferability of the proposed architecture beyond OCT5k, we conducted an additional evaluation on the independent Chiu OCT dataset [23]. This dataset contains retinal OCT B-scans acquired from eyes with diabetic macular edema and provides expert annotations for retinal layer structures. Because the original annotation scheme differs from that of OCT5k, the available boundary labels were mapped to the common region-based representation used in this study: background, ILM-region, OPL-region, IS-OS-region, IBRPE-region, and OBRPE-region.

Two complementary validation settings were considered. First, in the zero-shot setting, the RetiQueryNet model trained on OCT5k was directly evaluated on Chiu images without any parameter updates. This setting was used to assess direct cross-dataset transfer under domain shift. Second, in the fine-tuning setting, the OCT5k-pretrained model was adapted to the Chiu dataset using seed 42 under a patient-level split protocol. This setting was used to assess whether the proposed architecture can be effectively transferred to an independent OCT domain after task-specific adaptation. Since boundary-distance values were not directly comparable across the two annotation and preprocessing pipelines, external validation was assessed using mDice, mIoU, and class-wise Dice scores.

## 3. Experiments and Results

We evaluated RetiQueryNet on the OCT5k retinal layer segmentation task and compared it against six strong baseline models: U-Net [24], U-Net++ [25], FPN [26], MAnet [27], DeepLabV3 [28], and SegFormer-B0 [7]. All models were trained and evaluated under the same protocol, and the reported values correspond to the mean and standard deviation across multiple random seeds. We report mean Dice (mDice), mean IoU (mIoU), and mean surface distance (MSD) to jointly assess overlap quality and boundary precision.

### 3.1. Comparison with Baseline Models

Table 3 shows the overall comparison between RetiQueryNet and the baseline models. Among the baselines, SegFormer-B0 was the strongest model, achieving 0.9245 ± 0.0040 mDice, 0.8711 ± 0.0051 mIoU, and 1.1277 ± 0.0452 mean MSD. The proposed RetiQueryNet outperformed all baseline methods and achieved the best result for all three global metrics, with 0.9340 ± 0.0046 mDice, 0.8862 ± 0.0061 mIoU, and 0.9557 ± 0.0589 mean MSD.

Compared with the strongest baseline, RetiQueryNet improved mDice by 0.95 percentage points, improved mIoU by 1.51 percentage points, and reduced mean MSD by 0.1720 px. This reduction corresponds to a relative boundary improvement of roughly 15.3%. The gain is notable because it appears not only in overlap-based scores but also in the boundary-sensitive distance metric, which is particularly relevant in retinal OCT segmentation.

To further examine whether the improvement over the strongest baseline was consistent across repeated experimental runs, we performed an exploratory paired statistical comparison between RetiQueryNet and SegFormer-B0 using the three matched seed-wise evaluations. As summarized in Table 4, RetiQueryNet achieved higher mDice and mIoU and lower mean MSD in all three paired comparisons. The paired *t*-test yielded p-values below 0.05 for mDice, mIoU, and mean MSD, while the corresponding effect sizes were large in magnitude. Given the limited number of paired runs, these results are interpreted cautiously as supportive evidence of consistent performance gains rather than definitive population-level statistical inference.

### 3.2. Per-Layer Analysis

A more detailed view is given in Table 5, which reports the per-layer performance of RetiQueryNet. The model achieved near-perfect performance on ILM, with 0.9966 ± 0.0004 Dice and 0.4123 ± 0.0428 MSD. Strong results were also obtained for OPL and IS-OS, where Dice scores remained above 0.95. The more challenging lower retinal layers, IBRPE and OBRPE, showed lower overlap values, but the model still maintained strong performance with Dice scores close to 0.88 and sub-pixel to near-pixel boundary accuracy.

To show where the gain comes from, Table 6 compares the per-layer results of RetiQueryNet with those of the strongest baseline, SegFormer-B0. This improvement is evident in all layers, especially the deeper retinal layers. With respect to the IBRPE data set, there was an increase in the Dice score from 0.8657 to 0.8804, while the MSD decreased from 1.1039 to 0.9866. In the case of the OBRPE data set, the Dice score went up from 0.8548 to 0.8784, while the MSD improved from 1.0576 to 0.8982.

### 3.3. Ablation Study

To quantify the contribution of the main architectural components of RetiQueryNet, we performed a controlled ablation study using a fixed experimental setting with seed 42. Each ablated variant removes or modifies one design component while keeping the remaining training protocol unchanged. In particular, we evaluated the effects of removing the cross-attention module, removing the feed-forward refinement block, replacing learnable queries with fixed queries, replacing the structured query-driven head with a convolutional head, and freezing the encoder during training.

As shown in Table 7, the full RetiQueryNet configuration achieved the highest performance, with an mDice of 0.9411 and an mIoU of 0.8916. Removing cross-attention reduced the mDice to 0.9402, while removing the FFN block led to a further decrease to 0.9398. Fixed queries produced the lowest mDice among the query-based variants, reaching 0.9397, which supports the importance of learnable query embeddings. Replacing the proposed structured decoder with a conventional convolutional head also reduced performance to 0.9399. These results indicate that the gains of RetiQueryNet arise from the combined contribution of learnable queries, cross-attention, and structured query-based decoding rather than from a single isolated modification.

### 3.4. Seed-Wise Stability

The robustness of the proposed model is demonstrated by reporting the results obtained from each random seed in Table 8. The model performed consistently stable across all three trials. The best results were obtained using seed 123, which returned mDice of 0.9383, mIoU of 0.8912, and mean MSD of 0.9110 for RetiQueryNet. In the worst trial, the model still showed good global performance, suggesting that the proposed model does not depend on any one initialization or data split.

The same trend seen on the seed level is also applicable to the class level. Table 9 depicts the per layer scores on a per run basis. The ILM is always constant in all seed iterations, while the lower layers of the retina, specifically the IBRPE, tend to be more varied due to their thinner layering and lack of contrast.

### 3.5. Computational Efficiency Analysis

To assess the computational cost introduced by the proposed query-driven decoder, we compared RetiQueryNet with its SegFormer-B0 backbone in terms of model size, trainable parameters, floating-point operations, multiply–accumulate operations, inference latency, throughput, and peak GPU memory usage. The results are summarized in Table 10.

RetiQueryNet contains 4.28 million parameters and requires 14.06 GFLOPs, compared with 3.72 million parameters and 13.57 GFLOPs for SegFormer-B0. The additional decoder therefore increases the parameter count by approximately 15.1% and the computational cost by only 3.6%. Inference latency increases modestly from 6.08 ms to 6.37 ms per image, while throughput remains high at 157.08 FPS. Peak GPU memory rises from 174.29 MB to 190.78 MB. These findings show that the proposed query-based decoder introduces only limited computational overhead while preserving fast inference. When considered together with the accuracy gains reported in Table 3 and Table 4, this efficiency–accuracy trade-off supports the practical value of RetiQueryNet.

### 3.6. Sensitivity Analysis of the Loss Weight λ

To examine the effect of the loss balancing coefficient, we evaluated three values of *λ* in the combined objective function: 0.25, 0.50, and 0.75. These experiments were conducted under a fixed setting using seed 123, while all other training configurations were kept unchanged. The results are summarized in Table 11.

The model showed only marginal variation across the tested *λ* values. The mDice scores ranged from 0.9409 to 0.9411, while mIoU varied between 0.8912 and 0.8916. Layer-wise Dice values also remained highly stable across all settings. Although *λ* = 0.25 yielded the numerically highest mDice and mIoU, the differences were minimal. We retained *λ* = 0.50 in the main experiments because it provides an intuitively balanced contribution from cross-entropy and Dice loss, without introducing a meaningful reduction in performance.

### 3.7. External Validation on the Chiu Dataset

To evaluate the transferability of RetiQueryNet beyond the OCT5k benchmark, we conducted an additional experiment on the independent Chiu OCT dataset. Table 12 reports the results under two settings: direct zero-shot evaluation and fine-tuning on the external dataset.

In the zero-shot setting, RetiQueryNet achieved an mDice of 0.7188 and an mIoU of 0.5638. These results indicate that direct transfer from OCT5k to Chiu is challenging, likely because of differences in disease composition, image appearance, annotation protocol, and acquisition characteristics. Nevertheless, the model retained meaningful segmentation capability without being exposed to the target-domain data during training.

After fine-tuning on the Chiu dataset, performance increased substantially to an mDice of 0.8927 and an mIoU of 0.8090. The improvement was observed across all retinal layer regions, with particularly strong Dice scores for the ILM-region, OPL-region, and IS-OS-region. These findings suggest that, although direct cross-dataset generalization remains limited under domain shift, the proposed query-based architecture can be effectively adapted to an independent OCT dataset with a different clinical distribution.

### 3.8. Qualitative Results

**Figure 6:**
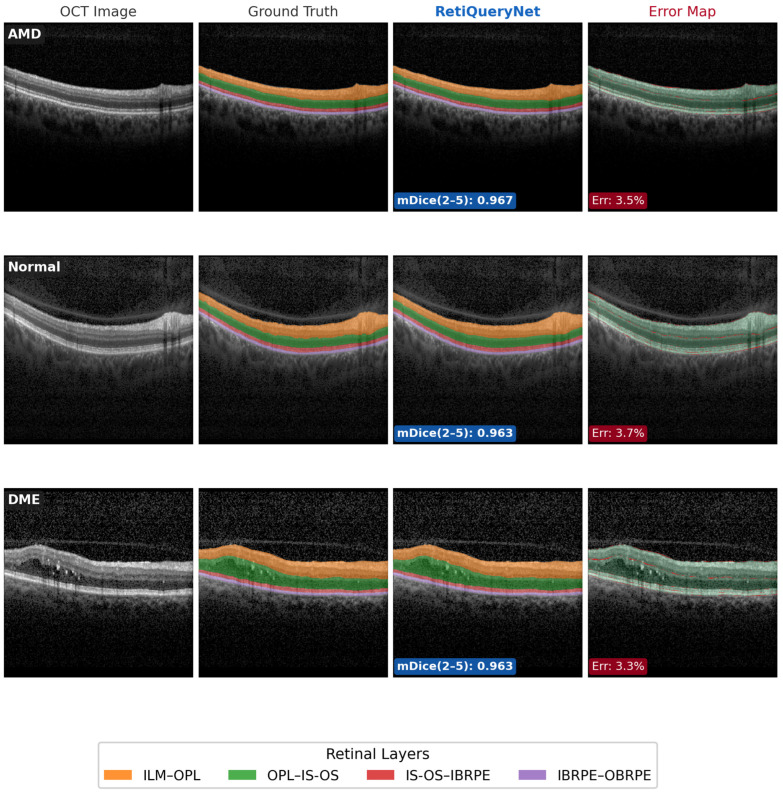
Qualitative segmentation results of RetiQueryNet on representative OCT images from AMD, Normal, and DME cases. Each row shows the input image, ground truth, predicted segmentation, and corresponding error map.

In order to further demonstrate the efficiency of the proposed model, qualitative results on some representative samples from OCT scans containing a wide range of clinical conditions such as AMD, DME, and normal samples are given. This is because these samples offer valuable information regarding the model performance for various anatomic and pathological deformations. It can be seen in Figure 6 that the generated segmentation outputs by RetiQueryNet closely follow the ground truth annotations with smooth and continuous retinal layers through the whole region. The samples shown in Figure 6 include the input OCT images, the ground truth masks, model prediction results, and error maps. It can be seen that the layers obtained by our model fit the structure of the retina well. For the case with retinal curvature caused by pathological changes, i.e., the AMD sample, the model shows its ability to adapt to this deformation and generate layers without any dislocations. With respect to the normal sample where the retinal layers present well-defined boundaries, the predicted results closely agree with the ground truth with little discrepancy in certain transition parts. With regard to the case containing fluid accumulation and local structural deformations, i.e., the DME sample, the model still shows its capability to generate stable layers in the right order.

**Figure 7:**
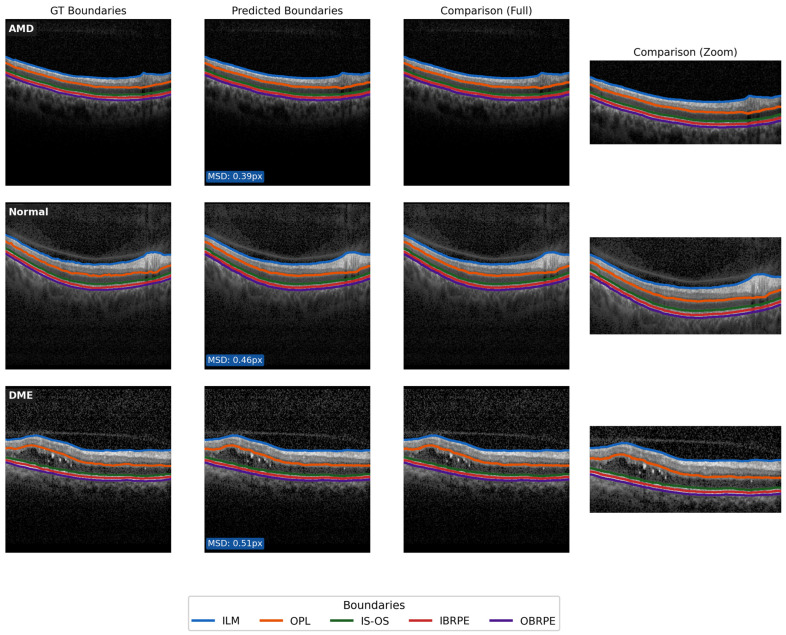
Boundary-level comparison between ground truth and predicted retinal layers. The figure includes full-resolution overlays and zoomed regions to highlight fine alignment differences.

In addition to analyzing the layer-level performance, we provide a visualization of the similarity between the predicted and true boundaries in Figure 7, which allows us to get an even better understanding of the model’s ability to predict small variations at boundaries. In Figure 7, the retinal layer boundaries for the ground truth and the prediction are shown on both the whole image scale and zoomed images. As can be seen from Figure 7, the predicted boundaries follow the reference ones accurately for all the layers. Furthermore, the values of MSD stay small, which indicates accurate localization of the boundaries. In terms of their anatomy, the model is close to perfect in predicting boundaries of the upper layers (ILM and OPL), since there exists a clear intensity transition between them. In lower layers, the difference is noticeable around the RPE area, where the intensity transition is smoother due to pathological changes. However, the boundaries obtained by our model stay regular and anatomically reasonable and do not contain impossible intersections.

### 3.9. Failure Case Analysis

**Figure 8:**
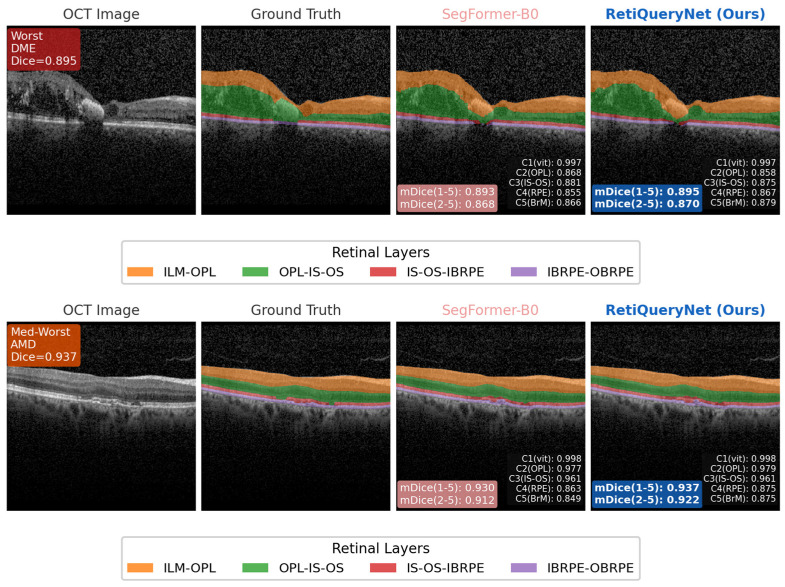
Representative failure cases of SegFormer-B0 and RetiQueryNet on challenging OCT images. Two difficult examples are shown: a severe DME case (**top**) and a moderately difficult AMD case (**bottom**). For each example, the figure presents the input OCT image, the ground-truth annotation, the SegFormer-B0 prediction, and the RetiQueryNet prediction. Both models experience performance degradation in regions with strong pathological deformation, low contrast, and boundary ambiguity. However, RetiQueryNet generally preserves smoother and more anatomically consistent retinal layer organization, particularly in the deeper retinal boundaries.

To better understand the limitations of the proposed model, we further analyzed challenging test samples in which the segmentation performance was lower than average. Representative failure cases are shown in Figure 8, including a severe DME case and a moderately difficult AMD case. These examples were selected to reflect clinically relevant situations with strong anatomical distortion, low local contrast, and boundary ambiguity.

As shown in Figure 8, the most difficult errors occur in regions where the retinal layers become highly compressed, irregular, or partially indistinguishable because of pathology-related deformation. In the severe DME example, both models struggle around the central distorted region, where the layer topology changes abruptly and the boundaries between OPL, IS-OS, and the deeper retinal interfaces become less distinct. Nevertheless, RetiQueryNet preserves a more coherent layer arrangement and produces slightly better overlap than SegFormer-B0, especially around the lower retinal boundaries.

A similar trend can be observed in the AMD example, where both methods show reduced accuracy near subtle structural irregularities and local boundary flattening. Even in this more difficult case, RetiQueryNet yields smoother and more anatomically consistent predictions, with visible improvements in the deeper layers such as IBRPE and OBRPE. These observations suggest that the proposed query-driven formulation improves structural stability under challenging conditions, although failure modes remain in cases with severe deformation, low contrast, or highly ambiguous boundary transitions.

Figure 8 highlights that the main remaining failure modes are associated with severe pathological distortion and reduced boundary visibility. In such cases, the upper and middle retinal layers may merge visually, while the lower boundaries become difficult to localize precisely. Compared with SegFormer-B0, RetiQueryNet still shows better structural continuity and fewer abrupt mask inconsistencies, but these examples confirm that highly abnormal scans remain challenging for both architectures.

## 4. Discussion

Results show consistent superiority of the proposed RetiQueryNet in terms of both region- and edge-based metrics. Even though the differences in numbers compared to the baselines may initially appear insignificant, closer analysis will reveal considerable improvements, especially in terms of structurally difficult areas. There is a notable difference in the ability of the method to retain structural integrity and predict realistic layer locations, especially in cases of pathologies like DME.

Some of the key strengths of the new model are related to the use of the queries for encoding of the retina layers. In contrast to traditional models using dense pixel-based classifications, this model uses an encoded representation of the layers that interacts with pixels through cross attention. As a result, each query can focus on a certain structure, making the whole process more stable. This feature allows for achieving increased stability and robustness to noise in comparison to the typical encoder–decoder paradigm.

The quantitative results confirm this notion as well. Improvements in the algorithm are more evident when dealing with thick and thin retinas, IBRPE, and OBRPE since there are several existing baseline algorithms that face challenges owing to issues related to low contrast and weak boundaries. RetiQueryNet not only maintains high Dice scores but achieves lower boundary errors as well, thereby indicating better spatial accuracy. The qualitative results are additional proof in this regard as the boundaries follow the ground truth despite curvature differences.

From a clinical perspective, improved boundary localization may be relevant for more reliable downstream morphometric analysis. OCT-derived measurements of retinal thickness and layer integrity are widely used in the assessment and monitoring of retinal diseases, including diabetic macular edema and age-related macular degeneration. Even when a reduction in mean surface distance appears numerically small, improved boundary fidelity can support more consistent estimation of thin retinal structures, particularly in deeper layers where anatomical transitions are subtle and clinically informative. In this context, the observed reduction in mean surface distance suggests that RetiQueryNet may offer practical value for applications that depend on precise retinal layer delineation. However, dedicated studies linking these segmentation gains to clinical decision outcomes remain necessary.

A further important aspect is that of the consistency shown by different random seeds. The generally small values for the standard deviation seen in both the Dice and IoU metrics show that the model is stable and less sensitive to initialization. These characteristics are advantageous, particularly in scenarios such as medical imaging where stability and reliability are highly desired. Despite this strength, however, some weaknesses exist. It seems that the model shows slightly more variance when dealing with the deeper retinal layers, especially when dealing with noisy or low-contrast regions. This result is expected because such areas are difficult even for manual labeling. A further practical consideration is that the current implementation defines one query for each output class. This choice is well aligned with the OCT5k annotation protocol, where the task involves background and five retinal layer regions. In principle, the same framework can be adapted to datasets with different anatomical granularity by modifying the number of learnable queries and retraining the final decoding stage. Nevertheless, this flexibility was not experimentally evaluated in the present study and should be examined in future work on datasets with alternative layer definitions or broader retinal annotation schemes.

On balance, the findings suggest that structured query formulation of retinal layers is a worthwhile avenue for retinal layer segmentation compared to conventional methods. The combination of transformer features with query decoding produces predictions that are both precise and anatomically sound.

## 5. Conclusions

In our paper, we introduce RetiQueryNet, a query-based segmentation framework that enables retinal layer segmentation in OCT scans. In the proposed solution, learnable queries along with cross-attention mechanisms are used to effectively represent anatomic structures. Our network architecture combines the transformer encoder with a compact query-driven decoding module that adds only modest computational overhead relative to the SegFormer-B0 backbone.

As shown through numerous experiments, RetiQueryNet is able to outperform multiple robust baselines in terms of multiple evaluation metrics such as Dice coefficient, Intersection over Union (IoU), and mean surface distance. These gains are particularly evident for complex regions where accuracy in boundary localization and consistency are vital. Further support is provided by qualitative results proving that RetiQueryNet generates smooth and consistent layers under different diseases.

Aside from performance, the suggested method also brings another angle to image segmentation in the medical domain. The idea that the anatomical layers are perceived as queries, as opposed to being considered only as classes of pixels, makes the proposed solution less prone to typical artifacts.

Future directions can expand the current approach to 3D OCT volumes and leverage temporal or multi-scan information. Furthermore, including uncertainty quantification or manual tuning by clinicians might improve the utility of this approach for real-world applications.

## Figures and Tables

**Table 1:** Summary of the OCT5k subset used in this study.

Category	AMD	DME	Normal	Overall
Images	739	403	530	1672
Cases	21	19	20	60
Image size	512 × 512	512 × 512	512 × 512	512 × 512
Layer1 thickness (px)	163.58 ± 33.55	158.30 ± 38.12	157.64 ± 27.57	160.24 ± 33.08 *
Layer2 thickness (px)	34.24 ± 4.46	43.33 ± 7.30	37.62 ± 4.25	37.48 ± 5.34 *
Layer3 thickness (px)	24.89 ± 3.06	36.31 ± 12.16	25.44 ± 3.25	28.88 ± 6.16 *
Layer4 thickness (px)	9.04 ± 1.18	7.20 ± 2.55	9.08 ± 1.12	8.44 ± 1.62 *
Layer5 thickness (px)	8.05 ± 1.30	8.34 ± 1.33	7.88 ± 1.18	8.09 ± 1.27 *

* Overall values represent the pooled mean ± standard deviation calculated across all images.

**Table 2:** Training configuration used in all comparative experiments.

Setting	Value
Optimizer	AdamW
Initial learning rate	1 × 10^−3^
Weight decay	1 × 10^−4^
Scheduler	Cosine annealing
Epochs	50
Batch size	4
Mixed precision	Yes
Model selection	Best validation Dice
Data split	Patient-level, 70/15/15
Seeds	42, 123, 456

**Table 3:** Overall comparison with baseline models.

Model	Seeds	mDice (Mean ± Std)	mIoU (Mean ± Std)	Mean MSD (Mean ± Std)	Best Val Dice (Mean ± Std)
DeepLabV3-ResNet50 [28]	2	0.9193 ± 0.0020	0.8645 ± 0.0033	1.2055 ± 0.0357	0.9218 ± 0.0114
FPN-EffB0	3	0.9235 ± 0.0038	0.8694 ± 0.0046	1.1840 ± 0.1243	0.9259 ± 0.0103
MAnet-EffB0	3	0.9216 ± 0.0026	0.8670 ± 0.0025	1.3391 ± 0.1007	0.9253 ± 0.0106
U-Net-EffB0	3	0.9229 ± 0.0033	0.8686 ± 0.0035	1.2894 ± 0.1289	0.9255 ± 0.0102
U-Net++-EffB0	3	0.9238 ± 0.0034	0.8701 ± 0.0037	1.1525 ± 0.1446	0.9267 ± 0.0105
SegFormer-B0	3	0.9245 ± 0.0040	0.8711 ± 0.0051	1.1277 ± 0.0452	0.9276 ± 0.0103
RetiQueryNet (ours)	3	0.9340 ± 0.0046	0.8862 ± 0.0061	0.9557 ± 0.0589	0.9340 ± 0.0132

**Table 4:** Direct performance and exploratory paired statistical comparison between SegFormer-B0 and RetiQueryNet across three matched seed-wise runs.

Metric	SegFormer-B0 Mean	RetiQueryNet Mean	Mean Paired Difference	95% CI of Difference	Paired *t*-Test *p*-Value	Cohen’s (d_z_)
mDice	0.9245	0.9340	+0.0095	[0.0053, 0.0138]	0.0106	5.562
mIoU	0.8711	0.8862	+0.0151	[0.0091, 0.0211]	0.0083	6.293
Mean MSD	1.1277	0.9557	−0.1720	[−0.2497, −0.0943]	0.0109	−5.497

Note: Statistical comparisons were performed on three matched seed-wise runs (seeds 42, 123, and 456). Negative differences in MSD indicate improved boundary localization for RetiQueryNet. Owing to the limited number of paired runs, these statistical results should be interpreted as exploratory evidence supporting the consistency of the observed improvements.

**Table 5:** Per-layer performance of RetiQueryNet.

Layer	Dice (Mean ± Std)	IoU (Mean ± Std)	MSD (Mean ± Std)
ILM	0.9966 ± 0.0004	0.9933 ± 0.0007	0.4123 ± 0.0428
OPL	0.9629 ± 0.0037	0.9330 ± 0.0042	1.2353 ± 0.0582
IS-OS	0.9516 ± 0.0026	0.9137 ± 0.0020	1.2460 ± 0.0386
IBRPE	0.8804 ± 0.0220	0.8010 ± 0.0255	0.9866 ± 0.2308
OBRPE	0.8784 ± 0.0036	0.7900 ± 0.0043	0.8982 ± 0.0370

**Table 6:** Per-layer comparison between SegFormer-B0 and RetiQueryNet.

Layer	Metric	SegFormer-B0	RetiQueryNet	Improvement
ILM	Dice	0.9952 ± 0.0007	0.9966 ± 0.0004	+0.0014
ILM	IoU	0.9906 ± 0.0013	0.9933 ± 0.0007	+0.0027
ILM	MSD	0.6349 ± 0.1135	0.4123 ± 0.0428	−0.2226
OPL	Dice	0.9601 ± 0.0042	0.9629 ± 0.0037	+0.0028
OPL	IoU	0.9279 ± 0.0049	0.9330 ± 0.0042	+0.0051
OPL	MSD	1.3539 ± 0.0859	1.2353 ± 0.0582	−0.1186
IS-OS	Dice	0.9466 ± 0.0032	0.9516 ± 0.0026	+0.0050
IS-OS	IoU	0.9043 ± 0.0034	0.9137 ± 0.0020	+0.0094
IS-OS	MSD	1.4878 ± 0.2460	1.2460 ± 0.0386	−0.2418
IBRPE	Dice	0.8657 ± 0.0175	0.8804 ± 0.0220	+0.0147
IBRPE	IoU	0.7774 ± 0.0201	0.8010 ± 0.0255	+0.0236
IBRPE	MSD	1.1039 ± 0.2093	0.9866 ± 0.2308	−0.1173
OBRPE	Dice	0.8548 ± 0.0049	0.8784 ± 0.0036	+0.0236
OBRPE	IoU	0.7552 ± 0.0046	0.7900 ± 0.0043	+0.0348
OBRPE	MSD	1.0576 ± 0.0175	0.8982 ± 0.0370	−0.1594

**Table 7:** Ablation analysis of RetiQueryNet components using seed 42.

Configuration	Description	mDice	mIoU
w/o Cross-Attention	Cross-attention module removed	0.9402	0.8900
w/o FFN Block	Query refinement FFN removed	0.9398	0.8892
Fixed Queries	Learnable queries replaced by fixed queries	0.9397	0.8891
Conv Head	Query-based structured decoder replaced by convolutional head	0.9399	0.8894
Frozen Encoder	Encoder weights frozen during training	0.9398	0.8894
Full RetiQueryNet	Complete proposed architecture	0.9411	0.8916

**Table 8:** Seed-wise performance of RetiQueryNet.

Seed	mDice	mIoU	Mean MSD	Best Val Dice	Best Epoch	Train	Val	Test
42	0.934466	0.888041	0.9337	0.918873	47	1174	255	243
123	0.938319	0.891190	0.9110	0.942510	45	1217	260	195
456	0.929155	0.879378	1.0224	0.940716	47	1150	303	219

**Table 9:** Seed-wise per-layer performance of RetiQueryNet.

Seed	Layer	Dice	IoU	MSD
42	ILM	0.996178	0.992533	0.4611
42	OPL	0.958724	0.928712	1.2821
42	IS-OS	0.948648	0.912937	1.2124
42	IBRPE	0.893152	0.817437	0.8231
42	OBRPE	0.875629	0.788585	0.8893
123	ILM	0.996881	0.993992	0.3944
123	OPL	0.965605	0.937165	1.1702
123	IS-OS	0.953663	0.915916	1.2375
123	IBRPE	0.893014	0.814006	0.8861
123	OBRPE	0.882432	0.794872	0.8665
456	ILM	0.996651	0.993395	0.3813
456	OPL	0.964515	0.933016	1.2536
456	IS-OS	0.952552	0.912197	1.2881
456	IBRPE	0.854924	0.771617	1.2507
456	OBRPE	0.877132	0.786667	0.9388

**Table 10:** Computational efficiency comparison between SegFormer-B0 and RetiQueryNet.

Model	Params (M)	Trainable Params (M)	GFLOPs	GMACs	Inference (ms/img)	P95 Latency (ms)	FPS	Peak GPU Mem (MB)
SegFormer-B0	3.72	3.72	13.57	6.79	6.08 ± 1.23	8.60	164.41	174.29
RetiQueryNet	4.28	4.28	14.06	7.03	6.37 ± 0.99	8.93	157.08	190.78

**Table 11:** Sensitivity analysis of the loss balancing coefficient λ.

λ	mDice	mIoU	Best Val Dice	ILM	OPL	IS-OS	IBRPE	OBRPE
0.25	0.9411	0.8916	0.9378	0.9971	0.9647	0.9558	0.9016	0.8865
0.50	0.9410	0.8913	0.9376	0.9970	0.9643	0.9552	0.9017	0.8865
0.75	0.9409	0.8912	0.9378	0.9971	0.9647	0.9548	0.9004	0.8874

**Table 12:** External validation performance of RetiQueryNet on the Chiu OCT dataset using seed 42.

Mode	Training/Adaptation Setting	mDice	mIoU	ILM-Region Dice	OPL-Region Dice	IS-OS-Region Dice	IBRPE-Region Dice	OBRPE-Region Dice
Zero-shot	Trained on OCT5k; evaluated directly on Chiu	0.7188	0.5638	0.8093	0.6602	0.7470	0.6980	0.6793
Fine-tuned	OCT5k-pretrained; fine-tuned on Chiu	0.8927	0.8090	0.9483	0.9191	0.9084	0.8605	0.8274

## Data Availability

The data analyzed in this study are publicly available. The OCT5k dataset used in this work can be accessed through the public repository associated with the original dataset publication: https://www.nature.com/articles/s41597-024-04259-z (accessed on 28 April 2026). No new clinical data were generated in this study. Additional experimental outputs and implementation details are available from the corresponding author upon reasonable request.

## Abbreviations

The following abbreviations are used in this manuscript:

OCT	Optical Coherence Tomography
DME	Diabetic Macular Edema
MSD	Mean Surface Distance
AMD	Age-related Macular Degeneration
CNN	Convolutional Neural Network
ILM	Inner Limiting Membrane
OPL	Outer Plexiform Layer
IS-OS	Inner Segment—Outer Segment
IBRPE	Inner Boundary of Retinal Pigment Epithelium
OBRPE	Outer Boundary of Retinal Pigment Epithelium
